# Degenerate codon mixing for PCR-based manipulation of highly repetitive sequences

**DOI:** 10.1186/s13104-018-3298-5

**Published:** 2018-03-27

**Authors:** Dhanushika Ratnayake, Morgan Newman, Michael Lardelli

**Affiliations:** 1 School of Biological Sciences, Alzheimer’s Disease Genetics Laboratory, University of Adelaide, North Terrace, Adelaide, SA 5005 Australia; 2 Present Address: Australian Regenerative Medicine Institute, Monash University, Wellington Road, Clayton, 3800 Australia

**Keywords:** Polyglutamine repeats, Codon redundancy, Degeneracy of the genetic code, PCR based repeat region amplification, Aggregate proteins, Autophagy

## Abstract

**Objective:**

Repeat expansion of polyglutamine tracks leads to a group of inherited human neurodegenerative disorders. Studying such repetitive sequences is required to gain insight into the pathophysiology of these diseases. PCR-based manipulation of repetitive sequences, however, is challenging due to the absence of unique primer binding sites or the generation of non-specific products.

**Results:**

We have utilised the degeneracy of the genetic code to generate a polyglutamine sequence with low repeat similarity. This strategy allowed us to use conventional PCR to generate multiple constructs with approximately defined numbers of glutamine repeats. We then used these constructs to measure the in vivo variation in autophagic degradation activity related to the different numbers of glutamine repeats, providing an example of their applicability to study repeat expansion diseases. Our simple and easily generalised method of generating low repetition DNA sequences coding for uniform stretches of amino acid residues provides a strategy for generating particular lengths of polyglutamine tracts using standard PCR and cloning protocols.

**Electronic supplementary material:**

The online version of this article (10.1186/s13104-018-3298-5) contains supplementary material, which is available to authorized users.

## Introduction

The aberrant expansion of unstable CAG repeats coding for polyglutamine (polyQ) tracts underlies a group of neurodegenerative diseases, including Huntington’s disease (HD) and several forms of spino-cerebellar ataxia (SCA) [1]. These diseases exhibit polyQ length-dependent toxicity, whereby age at disease onset is inversely correlated to the number of polyQ repeats [2]. They display common cellular and molecular mechanisms including protein aggregation and inclusion body formation [3]. Such protein aggregates depend strongly on autophagy for their clearance and dysfunction of this pathway may contribute to the pathology of these diseases [4]. Enhancement of autophagy has been suggested to have possible therapeutic value in such diseases showing protein aggregation by promoting the clearance of these aggregates and protecting cells against their toxic effects [5, 6]. However, studying the influence of polyQ tract length on aggregation kinetics is challenging due to difficulties faced when cloning repetitive DNA sequences primarily due to the lack of unique primer binding sites [7, 8]. Previously, several polymerase chain reaction (PCR) based methods to amplify repetitive DNA regions have been described [9–11]. However, most of these either generate nonspecific products, flawed repeats, or a collection of clones with varying numbers of repeats making the identification and isolation of the specific clone of interest laborious [12–14]. In order to investigate the autophagic degradation activity or ‘autophagic flux’ of polyQ protein aggregates we sought to clone reporter constructs containing more closely defined numbers of glutamine residues. We designed a polyQ sequence with low repeat similarity by exploiting the codon redundancy of the genetic code. This strategy allowed us to amplify close to the desired numbers of glutamine repeats (although still with some variability due to two distinct causes), which we subsequently used to assess in vivo variations in ‘autophagic flux’ in a larval zebrafish model for Alzheimer’s disease [15].

## Main text

### Results and discussion

**Fig. 1:**
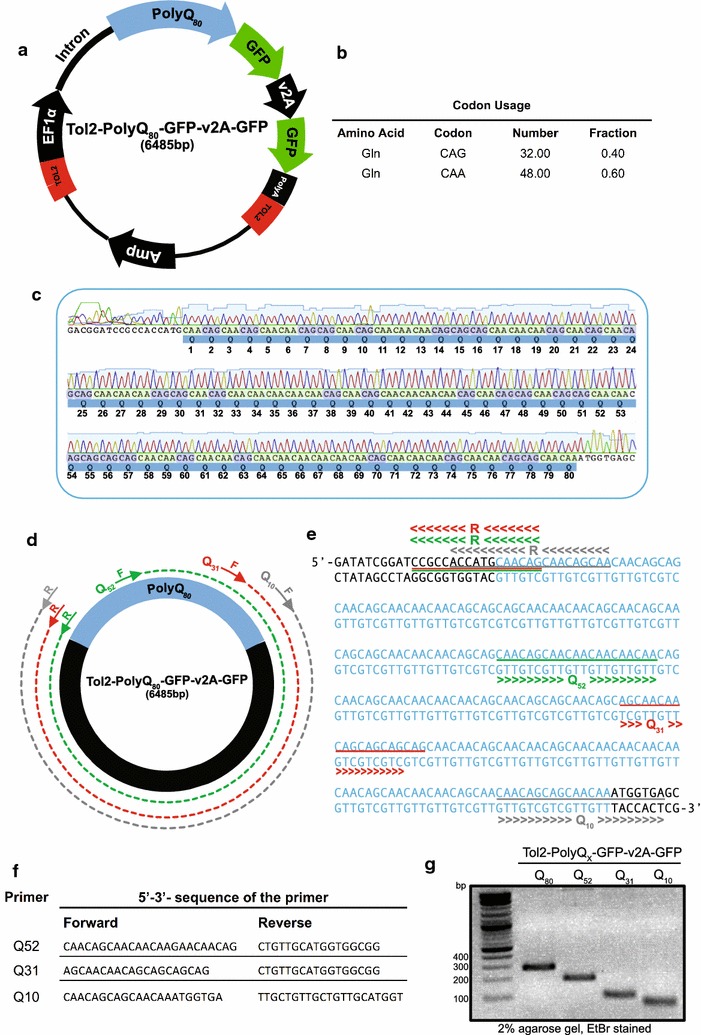
Generating the putative Q_52_, Q_31_ and Q_10_-GFP constructs using the Tol2-Q_80_-GFP-v2A-GFP multicistronic reporter construct. **a** Vector map of the Tol2-Q_80_-GFP-v2A-GFP construct. **b** Summary of CAA and CAG degenerate codon usage in this construct. **c** Chromatograph of the construct depicting the randomly interspaced glutamine coding CAG and CAA triplets. **d** Schematic illustration of PCR-based exclusion amplification of the Q_80_-GFP containing construct to generate polyQ constructs with lower numbers of glutamine repeats. **e** Q_80_ sequence showing Q_52_, Q_31_ and Q_10_ primer binding sites. **f** Sequences of primers intended to generate Q_52_, Q_31_ and Q_10_ constructs. **g** Analytical agarose gel electrophoresis for each of the putative Q_80_, Q_52_, Q_31_ and Q_10_ vectors using primers flanking the polyQ region. PCR product sizes of ~ 270, ~ 180, ~ 120 and ~ 60 bp for the intended putative Q_80_, Q_52_, Q_31_ and Q_10_-GFP constructs, respectively, were seen

The ‘autophagic flux’ assay described in Jiang et al. [15] is a quantitative green fluorescent protein (GFP) reporter assay that measures the ratiometric changes of polyQ-GFP to free GFP via Western blot analysis. A multicistronic reporter construct was designed to code for two proteins; polyQ linked to N-terminal GFP and free GFP. The viral 2A (v2A) sequence was placed as a linker region between the sequences coding for the two proteins to enable stoichiometric translation of two separate proteins from one open reading frame (Fig. 1a) [16].

We designed a polyQ sequence containing 80 glutamine repeats (Q_80_). The sequence was designed to have low repeat similarity by randomly interspacing glutamine-coding CAG triplets with glutamine-coding CAA triplets (Fig. 1b, c). The nucleotide substitutions were made by eye to generate a semi random pattern. This non-repetitive sequence design should not only enhance sequence stability during propagation in bacteria but also enabled the design of PCR primers that annealed to specific regions of the sequence.

The Q_80_-GFP-v2A-GFP construct described above was commercially synthesised (Biomatik Corporation) (see Additional file 1) and sub-cloned via the *BamH*I and *Cla*I restriction sites into the Tol2 transposon-based, pT2AL200R150G gene transfer vector (hereafter referred to as Tol2) available from the Kawakami laboratory [17] (Fig. 1a and see Additional file 2).

**Fig. 2:**
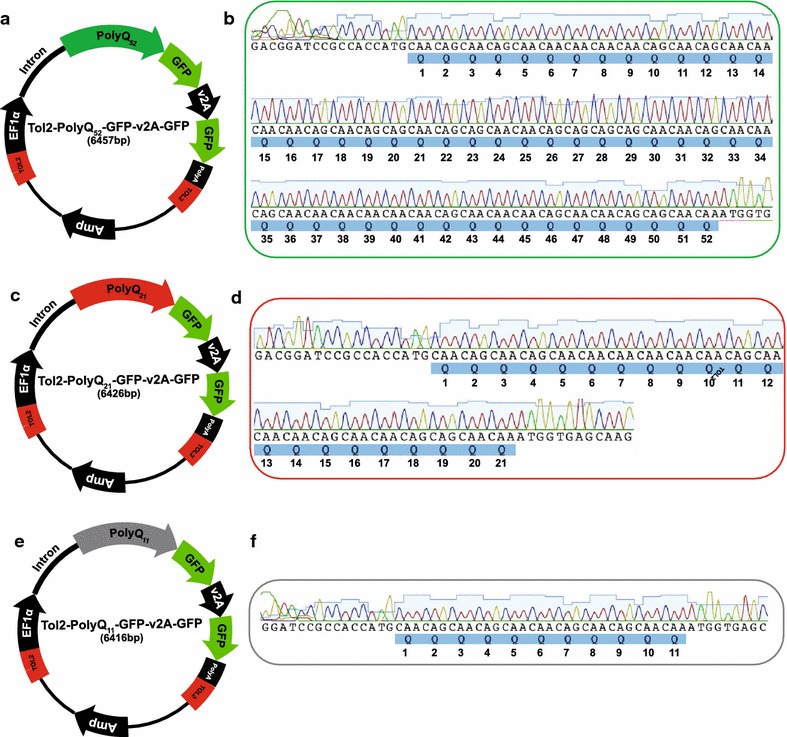
Vector map and sequence analysis of the constructs coding for Q_52_, Q_21_ and Q_11_-GFP generated by PCR based excision amplification. **a** Vector map of Tol2-Q_52_-GFP-v2A-GFP construct. **b** Chromatograph of the Q_52_ construct. **c** Vector map of Tol2-Q_21_-GFP-v2A-GFP construct. **d** Chromatograph of the Q_21_ construct. **e** Vector map of Tol2-Q_11_-GFP-v2A-GFP construct. **f** Chromatograph of the Q_11_ construct

PolyQ constructs with lower numbers of glutamine repeats were generated by PCR-based exclusion amplification of the Tol2-Q_80_-GFP-v2A-GFP construct. The primers were designed to amplify around the vector excluding a defined number of glutamine repeats to generate the constructs of interest (Fig. 1d–f). We aimed to generate vectors with approximately 52 (Q_52_), 31 (Q_31_) and 10 (Q_10_) glutamine repeats. The putative Q_52_ and Q_31_ vectors were generated using the same reverse primer coupled with different forward primers. This common reverse primer amplified 2 glutamine repeats, while the forward primers amplified the additional 50 and 29 glutamine repeats needed to generate the Q_52_ and Q_31_ vectors, respectively. The position of the reverse primer was shifted slightly to optimise the amplification of the putative Q_10_ vector, such that the reverse primer now amplified 5 glutamine repeats while the forward primer amplified the remaining 5 glutamine repeats (Fig. 1d–f). By using stringent annealing temperatures in the PCR reaction we obtained specific primer binding. Gel extracted and purified PCR products were phosphorylated, circularised by self-ligation and subsequently transformed into competent cells (see Additional file 3). PCR using primers that flanked the polyQ region showed approximately expected product sizes for the intended putative Q_52_, Q_31_ and Q_10_ vectors (Fig. 1g). The generated constructs were sequenced to determine whether the expected polyQ repeat numbers were present. While the Q_52_ construct had the expected number of glutamine repeats (Fig. 2a, b), sequencing revealed minor discrepancies with expected polyQ numbers for the other two constructs, where the Tol2-Q_31_-GFP-v2A-GFP vector had 21-glutamine repeats (hereafter referred to as Tol2-Q_21_-GFP-v2A-GFP) (Fig. 2c, d) and the Tol2-Q_10_-GFP-v2A-GFP vector had 11-glutamine repeats (hereafter referred to as Tol2-Q_11_-GFP-v2A-GFP) (Fig. 2e, f). Further analysis revealed that the generated Q_21_ sequence was derived directly from the original sequence and the loss of glutamine repeats was due to the Q_31_ forward primer binding 30 bp downstream from the predicted binding site. In contrast, the additional glutamine repeat in the Q_11_ sequence was generated de novo, an addition of a CAA codon.

**Fig. 3:**
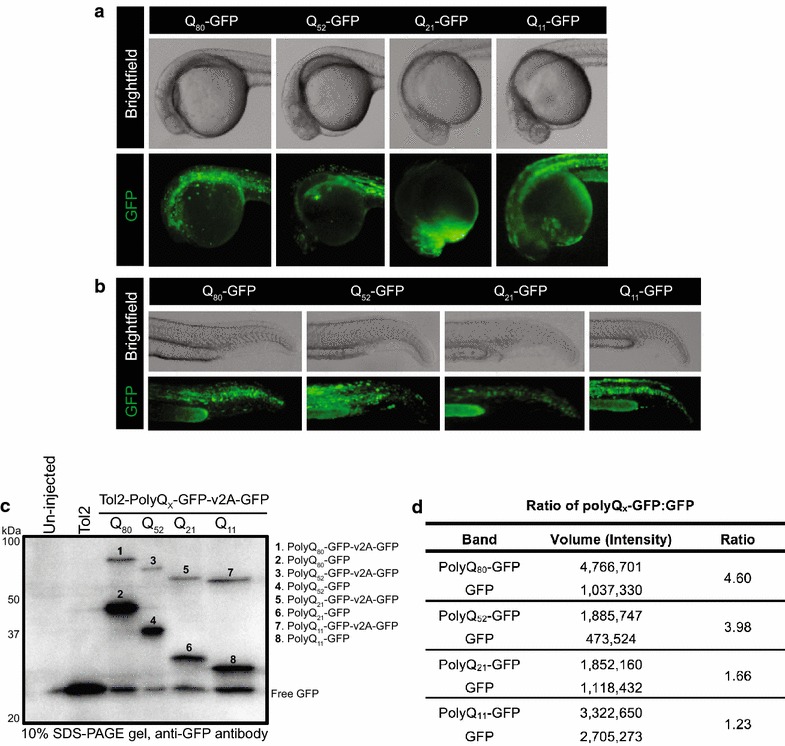
Analysis of the Tol2-Q_X_-GFP-v2A-GFP construct expression in *D. rerio.* Zebrafish embryos were injected with 25 ng/µL of the Tol2-Q_X_-GFP-v2A-GFP construct and 25 ng/µL mRNA coding for Tol2 transposase. **a** Brightfield (top panels) and fluorescent (bottom panels) images of the left side view of zebrafish embryo head and trunk regions expressing Q_80_, Q_52_, Q_21_ and Q_11_-GFP, ~ 24 hpf. **b** Brightfield (top panels) and fluorescent (bottom panels) images of the left side view of zebrafish embryo trunk and tail regions expressing Q_80_, Q_52_, Q_21_ and Q_11_-GFP, ~ 24 hpf. **c** Western blot analysis of the expression of Q_x_-GFP constructs in *D. rerio*. Proteins were isolated from ~ 24 hpf embryos expressing the Q_80_, Q_52_, Q_21_ and Q_11_-GFP constructs. The proteins were resolved on a 10% SDS-PAGE gel and transferred to a nitrocellulose membrane. The membrane was probed with an anti-GFP antibody. The “empty” Tol2 vector possesses an expressed GFP gene that is replaced during construct insertion. **d** Western blot quantification. The GFP intensity for each numbered band of the Western blot and the Q_x_-GFP to free GFP ratio are presented

To study the aggregation kinetics and ‘autophagic flux’ of polyQ protein in vivo, we injected the generated polyQ vectors (25 ng/μL) and transposase mRNA (25 ng/μL) into groups of one-cell-stage zebrafish embryos (Fig. 3a, b). Western blot analysis with anti-GFP antibody of 24 h post fertilisation (hpf) embryo lysates (10 embryos per sample) was carried out for each group. The empty Tol2 vector (25 ng/μL) and transposase mRNA (25 ng/μL) injected and un-injected embryos were included as controls. Embryos injected with the polyQ constructs produced two bands detected by the anti-GFP antibody as expected; GFP attached to polyQ (polyQ_80_-GFP at ~ 48 kDa, polyQ_52_-GFP at ~ 38 kDa, polyQ_21_-GFP at ~ 33 kDa and polyQ_11_-GFP at ~ 31 kDa), and free GFP (~ 27 kDa) (Fig. 3c). Each of the polyQ-GFP construct expressing embryo lysates also showed a fainter band of higher protein size corresponding to the full length polyQ_X_-GFP-v2A-GFP construct (polyQ_80_-GFP-v2A-GFP at ~ 75 kDa, polyQ_52_-GFP-v2A-GFP at ~ 65 kDa, polyQ_21_-GFP-v2A-GFP at ~ 60 kDa, and polyQ_11_-GFP-v2A-GFP at ~ 58 kDa). This band represents the ~ 10% of the total protein that is translated as a single, full-length protein when using the v2A sequence system [18]. The Q_80_-GFP:GFP, Q_52_-GFP:GFP, Q_21_-GFP:GFP and Q_11_-GFP:GFP ratios are ~ 5, ~ 4, ~ 2 and ~ 1, respectively (Fig. 3d). As the v2A sequence allows for the stoichiometric translation of the polyQ-GFP and GFP proteins, in theory the polyQ-GFP:GFP ratio should be 1. The greater ratios observed may indicate an accumulation of those proteins. These observations are in agreement with the literature, where it has been shown that polyQ-GFP fusion constructs containing greater than 19 glutamine residues aggregate within transfected cells in a length-dependent manner [19]. These observations lead us to conclude that our Tol2-Q_X_-GFP-v2A-GFP constructs provide a useful tool to study ‘autophagic flux’ in vivo in a larval zebrafish model.

### Conclusion

In conclusion this study provides a robust and easily adoptable solution to generate close to intended lengths of polyQ repeats. In order to generate exact numbers of glutamine repeats subsequent rounds of amplifications with altered primer sequences can be carried out. In addition the primer length can be increased to enhance specificity of primer annealing to the template. Our technique has several advantages over the existing methods for PCR-based amplification of repetitive regions and aims to minimise the generation of non-specific PCR products and flawed repeats by exploiting the codon redundancy of the genetic code to generate a synonymous DNA coding sequence with reduced repetition. In addition, our approach is not limited to generating polyQ repeat sequences but can also be generalised to generate other nucleotide repeat sequences. Furthermore, our method is relatively cheap, as only the initial polyQ_80_-GFP-v2A-GFP construct requires commercial synthesis, the cost of which depends on the price per nucleotide base, length, purity and mass. All other materials required are standard reagents used for molecular cloning. In summary the technique described provides an easy to adopt, affordable solution to generate repeat-coding DNA sequences that can be manipulated as required.

## Limitations

- Initial construct requires commercial synthesis.
- May not generate the exact number of glutamine repeats required.

## Additional files

**Additional file 1.** Sequence design for the Q_80_-GFP-v2A-GFP construct. The commercially synthesised Q_80_-GFP-v2A-GFP construct is flanked by *BamH*I *I* and *Cla*I *I* restriction sites used for sub cloning into the desired final vector.

**Additional file 2.** Sub-cloning the Q_80_-GFP-v2A-GFP construct into the Tol2 vector. The Q_80_-GFP-v2A-GFP construct provided in the pBluescript II SK(+) vector is sub-cloned into the pT2AL200R150G (Tol2) vector.

**Additional file 3.** Exclusion amplification of the Tol2-Q_80_-GFP-v2A-GFP construct to generate constructs containing putative Q_52_, Q_31_ or Q_10_ repeats. Detailed method for generating polyQ constructs with lower number of glutamine repeats using the Tol2-Q_80_-GFP-v2A-GFP construct as a template.

## Abbreviations

Q_80_: 80 glutamine repeats
GFP: green fluorescent protein
hpf: hours post fertilisation
HD: Huntington’s disease
polyQ: polyglutamine
PCR: polymerase chain reaction
SCA: spino-cerebellar ataxia
v2A: viral 2A
