# Supplementary material for: Degenerate codon mixing for PCR-based manipulation of highly repetitive sequences
Source: BMC Res Notes. 2018 Mar 27;11:202. doi: 10.1186/s13104-018-3298-5 (PMC5870680; doi:10.1186/s13104-018-3298-5)
Supplement: Supplementary file 1 — Additional file 1. Sequence design for the Q80-GFP-v2A-GFP construct. The commercially synthesised Q80-GFP-v2A-GFP construct is flanked by BamHI I and ClaI I restriction sites used for sub cloning into the desired final vector. [file 13104_2018_3298_MOESM1_ESM.docx]

**Additional file 1: Sequence design for the Q_80_-GFP-v2A-GFP construct**

Different regions of the sequence are labeled with different highlighted colours corresponding to the legend below:

EcoRV-BamHI-Kozak-Start codon-polyQ_80_-GFP-v2A-GFP-Stop-ClaI-EcoRI-EcoRV

Sequence of the polyQ_80_-GFP-v2A-GFP construct:

5’-

GATATCGGATCCGCCACCATGCAACAGCAACAGCAACAACAGCAGCAACAGCAACAACAACAGCAGCAGCAACAACAACAGCAACAGCAACAGCAGCAACAACAACAGCAGCAACAGCAACAACAACAACAACAGCAACAGCAACAACAACAACAGCAACAGCAGCAACAGCAGCAACAACAGCAGCAGCAGCAACAACAGCAACAACAGCAACAACAACAACAACAACAGCAACAACAACAGCAACAACAGCAGCAACAAatggtgagcaagggcgaggagctgttcaccggggtggtgcccatcctggtcgagctggacggcgacgtaaacggccacaagttcagcgtgtccggcgagggcgagggcgatgccacctacggcaagctgaccctgaagttcatctgcaccaccggcaagctgcccgtgccctggcccaccctcgtgaccaccctgacctacggcgtgcagtgcttcagccgctaccccgaccacatgaagcagcacgacttcttcaagtccgccatgcccgaaggctacgtccaggagcgcaccatcttcttcaaggacgacggcaactacaagacccgcgccgaggtgaagttcgagggcgacaccctggtgaaccgcatcgagctgaagggcatcgacttcaaggaggacggcaacatcctggggcacaagctggagtacaactacaacagccacaacgtctatatcatggccgacaagcagaagaacggcatcaaggtgaacttcaagatccgccacaacatcgaggacggcagcgtgcagctcgccgaccactaccagcagaacacccccatcggcgacggccccgtgctgctgcccgacaaccactacctgagcacccagtccgccctgagcaaagaccccaacgagaagcgcgatcacatggtcctgctggagttcgtgaccgccgccgggatcactctcggcatggacgagctgtacaagggctccggagctacaaatttctctctgttgaaacaggctggtgacgtcgaggagaatcctggcccaATGGTGAGCAAGGGAGAGGAGCTGTTCACAGGAGTGGTGCCTATCCTGGTGGAGCTGGACGGAGACGTGAACGGACACAAGTTCAGCGTGAGCGGAGAGGGAGAGGGAGACGCTACATACGGAAAGCTGACACTGAAGTTCATCTGTACAACAGGAAAGCTGCCTGTGCCTTGGCCTACACTGGTGACAACACTGACATACGGAGTGCAGTGTTTCAGCAGATACCCTGACCACATGAAGCAGCACGACTTCTTCAAGAGCGCTATGCCTGAGGGATACGTGCAGGAGAGAACAATCTTCTTCAAGGACGACGGAAACTACAAGACAAGAGCTGAGGTGAAGTTCGAGGGAGACACACTGGTGAACAGAATCGAGCTGAAGGGAATCGACTTCAAGGAGGACGGAAACATCCTGGGACACAAGCTGGAGTACAACTACAACAGCCACAACGTGTACATCATGGCTGACAAGCAGAAGAACGGAATCAAGGTGAACTTCAAGATCAGACACAACATCGAGGACGGAAGCGTGCAGCTGGCTGACCACTACCAGCAGAACACACCTATCGGAGACGGACCTGTGCTGCTGCCTGACAACCACTACCTGAGCACACAGAGCGCTCTGAGCAAGGACCCTAACGAGAAGAGAGACCACATGGTGCTGCTGGAGTTCGTGACAGCTGCTGGAATCACACTGGGAATGGACGAGCTGTACAAGtagatcgatgaattcGATATC-3’
