# Supplementary material for: Degenerate codon mixing for PCR-based manipulation of highly repetitive sequences
Source: BMC Res Notes. 2018 Mar 27;11:202. doi: 10.1186/s13104-018-3298-5 (PMC5870680; doi:10.1186/s13104-018-3298-5)
Supplement: Supplementary file 2 — Additional file 2. Sub-cloning the Q80-GFP-v2A-GFP construct into the Tol2 vector. The Q80-GFP-v2A-GFP construct provided in the pBluescript II SK(+) vector is sub-cloned into the pT2AL200R150G (Tol2) vector. [file 13104_2018_3298_MOESM2_ESM.docx]

**Additional file 2: Sub-cloning the** **Q_80_-GFP-v2A-GFP construct into the Tol2 vector**

The pT2AL200R150G (Tol2) vector (~5.5 kbp) contains a GFP expression cassette flanked by *BamH* I and *Cla* I restriction sites. The GFP expression cassette (~700 bp) was removed through dual restriction enzyme cleavage using *BamH* I HF *^®^* and *Cla* I (NEB, R3136S and R0197S respectively).

The Q_80_-GFP-v2A-GFP sequence was originally provided in the pBluescript II SK(+) vector. The Q_80_-GFP-v2A-GFP region (~1.8 kbp) was excised from the vector using restriction enzyme cleavage with *BamH* I HF *^®^* and *Cla* I (NEB, R3136S and R0197S respectively), and then ligated into the Tol2 backbone (~4.7 kbp) using T4 DNA ligase (Sigma-Aldrich, KEM0020).
