# Supplementary material for: Degenerate codon mixing for PCR-based manipulation of highly repetitive sequences
Source: BMC Res Notes. 2018 Mar 27;11:202. doi: 10.1186/s13104-018-3298-5 (PMC5870680; doi:10.1186/s13104-018-3298-5)
Supplement: Supplementary file 3 — Additional file 3. Exclusion amplification of the Tol2-Q80-GFP-v2A-GFP construct to generate constructs containing putative Q52, Q31 or Q10 repeats. Detailed method for generating polyQ constructs with lower number of glutamine repeats using the Tol2-Q80-GFP-v2A-GFP construct as a template. [file 13104_2018_3298_MOESM3_ESM.docx]

**Additional file 3: Exclusion amplification of the Tol2-Q_80_-GFP-v2A-GFP construct to generate constructs containing putative Q_52_, Q_31_ or Q_10_ repeats**

PCRs on the Tol2-Q_80_-GFP-v2A-GFP construct were performed as 20 μL reactions using Phusion^®^ High-Fidelity DNA polymerase (New England BioLabs^®^ *Inc*.). The primers 5’-CAACAGCAACAACAAGAACAACAG-3’ and 5’-CTGTTGCATGGTGGCGG-3’ to generate the Q_52_ construct, 5’-AGCAACAACAGCAGCAGCAG-3’ and 5’-CTGTTGCATGGTGGCGG-3’ to generate the Q_31_ construct and the primers 5’-CAACAGCAGCAACAAATGGTGA-3’ and 5’- TTGCTGTTGCTGTTGCATGGT-3’ to generate the Q10 construct were used. The PCRs had a final concentration of 1X Phusion^®^ HF buffer, 200 µM dNTPs, 10 µM/µL forward primer, 10 µM/µL reverse primer, 20 ng/µL template DNA and 0.02 U/µL Phusion^®^ High-Fidelity DNA polymerase. The PCR reaction conditions consisted of an initial denaturation step at 98^0^C for 30 seconds, followed by 15 cycles of denaturation at 98^0^C for 10 seconds, annealing at 68°C for Q_52_ and Q_31_ and at 70°C for Q_10_ for 20 seconds and extension at 72^0^C for 3 min 20 sec for Q_52_ and Q_31_ and for 3 min for Q_10_ followed by a final elongation step at 72^0^C for 5 minutes. For analysis, 6X gel loading dye (New England BioLabs^®^ *Inc*.), to give a final concentration of 1X) was added to the PCR samples and loaded into a 1% (w/v) agarose gel in Tris Acetate-EDTA (TAE) buffer containing 1mg/mL ethidium bromide (Sigma-Aldrich^®^). Electrophoresis was carried out at 90V and 500mA for 1 hour. The ~6.4 kb, ~6.3 kb and ~6.2 kb bands corresponding to linear putative Q_52_, Q_31_ and Q_10_ GFP constructs in Tol2, respectively were purified by gel extraction using the QIAquick Gel Extraction Kit protocol using a microcentrifuge (QIAGEN^®^). Phosphorylation of the purified linear constructs was carried out using T4 polynucleotide kinase (New England Biolabs^®^) following the manufacture’s phosphorylation protocol. These were then circularized in 50µL reactions containing a final concentration of 10-50ng linear DNA, 1X T4 DNA ligase buffer and 5U of T4 DNA ligase (New England Biolabs^®^) for 1 hour and 30 minutes at room temperature followed by heat inactivation of T4 DNA ligase at 70^0^C for 5 minutes. The circularized vectors were transformed into competent DH5α cells using a standard heat-shock transformation protocol and plated on Luria broth agar plates containing ampicillin and incubated at 37^0^C overnight. The resulting colonies were screened for the respective Tol2-Q_X_-GFP-v2A-GFP construct by performing PCR on thermo-lysed bacteria using primers flanking the polyQ region (5’- TCCGCCACCATGCAACAG-3’ and 5’- GCCCTTGCTCACCATTTGTT-3’). An *E. coli* colony possessing a single clone of each of the constructs was isolated and amplified in liquid culture. Plasmid DNA was then isolated from these cultures and purified using the QIAprep Spin Miniprep Kit protocol (QIAGEN^®^).
